# Materia Medica and Therapeutics

**Published:** 1891-08

**Authors:** 


Materia Medica and Therapeutics, with especial reference to the Clinical Application of Drugs. By John V. Shoemaker, A. M., M. D., Professor of Materia Medica, Pharmacology, Therapeutics, and Clinical Medicine, and Clinical Professor of Diseases of the Skin in the Medico-Chirurgical College of Philadelphia; Physician to the Medico-Chirurgical Hospital, etc. Volume II. of a Treatise on Materia Medica, Pharmacology, and Therapeutics, being an independent volume upon drugs. 8vo, pp. xx.—625. Philadelphia and London: F. A. Davis. 1891. Price, cloth, $3.50; sheep, $4.50.

In reviewing Vol. I. of this treatise, the writer expressed the hope that Vol. II. would be equally as interesting and valuable, and now since the advent of the latter we have no hesitation in saying that for completeness, originality, and amount of information, we know of no book on materia medica to equal it. This second volume is in itself a complete treatise on the pharmacological, physiological and therapeutical action of the various drugs now used in medicine. Each drug is described in the following manner : Its Latin and English equivalents — are given with a full list of its preparations, together with their doses ; then follows a description of the drug and its use externally ; its physiological and toxicological actions are briefly reviewed, followed by its therapy. Numerous formulas accompany almost every drug, thus aiding the student in the art of prescription-writing, also in regard to its compatibilities and pleasant vehicles.

Among some of the newer remedies may be mentioned Koch's tuberculin ; the dose is given as 1-64 grain hypodermatically ; its value is summarized as follows : 1. It exercises a specific and selective action. 2. It is therefore a valuable means of diagnosis as to even the unsuspected existence of tuberculosis in the body. 3. Under its continued action tuberculous tissues are destroyed, and, when possible, are cast off by sloughing. 4. No case of permanent cure has to our knowledge been positively recorded. 5. The large amount of clinical experience already existing warrants the further trial and careful observation of the remedy in tuberculous cases. 6. Even should the future prove the lymph unsuited to permanently cure tuberculosis, the discovery will still remain an index for the future researches in the domain of scientific therapeutics.

An appendix containing formulas for hypodermatic medication adds much to the usefulness of the book.

The typography is similar to Vol. I. W. C. K.

